# The burden of lower respiratory infections and their underlying etiologies in the Middle East and North Africa region, 1990–2019: results from the Global Burden of Disease Study 2019

**DOI:** 10.1186/s12890-022-02301-7

**Published:** 2023-01-04

**Authors:** Ahad Ashrafi-Asgarabad, Saied Bokaie, Jamshid Razmyar, Hesameddin Akbarein, Seyed Aria Nejadghaderi, Kristin Carson-Chahhoud, Mark J. M. Sullman, Jay S. Kaufman, Saeid Safiri

**Affiliations:** 1 Divisions of Epidemiology and Zoonoses, Department of Food Hygiene and Quality Control, Faculty of Veterinary Medicine, University of Tehran, Tehran, Iran; 2 Department of Avian Diseases, Faculty of Veterinary Medicine, University of Tehran, Tehran, Iran; 3 School of Medicine, Shahid Beheshti University of Medical Sciences, Tehran, Iran; 4 Australian Centre for Precision Health, University of South Australia, Adelaide, South Australia Australia; 5 School of Medicine, University of Adelaide, Adelaide, South Australia Australia; 6 Department of Life and Health Sciences, University of Nicosia, Nicosia, Cyprus; 7 Department of Social Sciences, University of Nicosia, Nicosia, Cyprus; 8 Department of Epidemiology, Biostatistics, and Occupational Health, Faculty of Medicine, McGill University, Montreal, QC Canada; 9 Tuberculosis and Lung Diseases Research Center, Tabriz University of Medical Sciences, Tabriz, Iran; 10 Department of Community Medicine, Faculty of Medicine, Tabriz University of Medical Sciences, Tabriz, Iran

**Keywords:** Lower respiratory infection, Risk factor, Etiology, Global burden of disease, Middle East and North Africa

## Abstract

**Background:**

Lower respiratory infections (LRIs) cause substantial mortality and morbidity. The present study reported and analysed the burden of LRIs in the Middle East and North Africa (MENA) region between 1990 and 2019, by age, sex, etiology, and socio-demographic index (SDI).

**Methods:**

The data used in this study were sourced from the Global Burden of Disease (GBD) study 2019. The annual incidence, deaths, and disability-adjusted life-years (DALYs) due to LRIs were presented as counts and age-standardised rates per 100,000 population, along with their 95% uncertainty intervals (UIs). The average annual percent changes (AAPC) in the age-standardised incidence, death and DALYs rates were calculated using Joinpoint software and correlations (Pearson’s correlation coefficient) between the AAPCs and SDIs were calculated using Stata software.

**Results:**

In 2019, there were 34.1 million (95% UI 31.7–36.8) incident cases of LRIs in MENA, with an age-standardised rate of 6510.2 (95% UI 6063.6–6997.8) per 100,000 population. The number of regional DALYs was 4.7 million (95% UI 3.9–5.4), with an age-standardised rate of 888.5 (95% UI 761.1–1019.9) per 100,000 population, which has decreased since 1990. Furthermore, Egypt [8150.8 (95% UI 7535.8–8783.5)] and Afghanistan [61.9 (95% UI 52.1–72.6)] had the highest age-standardised incidence and death rates, respectively. In 2019, the regional incidence and DALY rates were highest in the 1–4 age group, in both females and males. In terms of deaths, pneumococcus and H. influenza type B were the most and least common types of LRIs, respectively. From 1990 to 2019, the burden of LRIs generally decreased with increasing SDI. There were significant positive correlations between SDI and the AAPCs for the age-standardised incidence, death and DALY rates (*p* < 0.05). Over the 1990–2019 period, the regional incidence, deaths and DALYs attributable to LRIs decreased with AAPCs of − 1.19% (− 1.25 to − 1.13), − 2.47% (− 2.65 to − 2.28) and − 4.21% (− 4.43 to − 3.99), respectively.

**Conclusions:**

The LRI-associated burden in the MENA region decreased between 1990 and 2019. SDI had a significant positive correlation with the AAPC and pneumococcus was the most common underlying cause of LRIs. Afghanistan, Yemen and Egypt had the largest burdens in 2019. Further studies are needed to investigate the effectiveness of healthcare interventions and programs to control LRIs and their risk factors.

**Supplementary Information:**

The online version contains supplementary material available at 10.1186/s12890-022-02301-7.

## Introduction

Respiratory infections have a high attributable mortality and morbidity, partially due to their high rate of transmission [1, 2]. These are the most common infections in developing countries, accounting for 20–40% of pediatric hospital visits, and are one of the most common causes of death in children under five years old [3].

In some cases, viral infections can lead to superimposed bacterial infections [4, 5]. Influenza is the most common viral cause that can also lead to a secondary bacterial infection. These infections can be upper and lower respiratory tract infections, which account for 5–27% of acute infections [6, 7]. In addition, infections caused by respiratory syncytial virus (RSV) can have different clinical presentations, such as rhinitis and bronchitis [8, 9].

There are many risk factors that can increase the LRI disease burden and morbidity, such as malnutrition, indoor and outdoor air pollution, smoking and chronic lung disease, as well as poor patient management [10]. Also, etiological factors such as haemophilus influenza type B (Hib), influenza, streptococcus pneumonia, and RSV can increase the attributable burden [11].

A number of studies have examined the burden of lower respiratory infections (LRIs) worldwide by etiology (i.e. streptococcus pneumonia, Hib, RSV, and influenza) using data from the Global Burden of Disease 2015 and 2016 projects, which are in need of updating [12, 13]. There has also been a recent global study which investigated age and sex differences in LRIs, along with their risk factors [14]. Nevertheless, to our best of knowledge there has been no comprehensive epidemiological study of LRIs in the Middle East and North Africa (MENA) region and the countries that comprise this region. Therefore, the present study used GBD 2019 data to report and analyse the burden of LRIs in the 21 countries and territories that make up the MENA region according to age, sex, etiology, and socio-demographic index (SDI) level, from 1990 to 2019.

## Methods

### Overview

We extracted data from the GBD database in April 2022. In this section, we briefly describe the methods and the processes used by GBD. The GBD is an international research programme that is conducted by the Institute of Health Metrics and Evaluation (IHME) at the University of Washington. The 2019 iteration collected information about 369 diseases and injuries and 87 risk factors in 204 countries and territories over the previous 30 years. A comprehensive explanation of the GBD 2019 methodology has been reported elsewhere [15] and all estimates are accessible online: https://vizhub.healthdata.org/gbd-compare/ and http://ghdx.healthdata.org/gbd-results-tool.

### Case definition and data sources

The LRI case definitions were clinician-diagnosed pneumonia or bronchiolitis, using the disease codes from version 10 of the International Classification of Diseases (ICD) (i.e., A48.1, J09-J22, J85.1, P23-P23.9, and U04) and version 9 (i.e., 073.0–073.6, 079.82, 466–469, 480–489, 513.0, and 770.0) [15]. The modeling of LRI etiology involved a separate process to that of estimating the overall LRI incidence and prevalence. The etiologies consisted of the following causal pathogens: influenza, RSV, streptococcus pneumonia, and Hib.

The data for estimating the incidence and prevalence of LRIs were obtained from several sources, including the GBD 2017 data, a systematic review of the literature, hospital inpatient and outpatient data, claims data, as well as surveys that were representative of the population. In terms of the LRI etiologies [15] population attributable fractions for influenza and RSV were extracted from a systematic review of the proportion of LRI cases that tested positive for each pathogen. In addition, information about Hib and streptococcus pneumonia (pneumococcal pneumonia) were sourced from a systematic review on vaccine efficacy. This included only studies that: had a sample size of 100 or more, had a study duration of at least one year, and had LRI case definitions (i.e., pneumonia or bronchiolitis). In total, 121 studies were identified, but only two studies met the strict inclusion criteria and were included. The exclusion criteria included those which only reported pandemic-related H1N1 influenza and those with a case definition of an influenza-like illness. In cases where the participants’ ages were not reported, the participants were assigned to age ranges based on the prevalence-weighted mean age of LRIs in the appropriate year/sex/location [15]. Furthermore, a systematic review, up to May 2017, was conducted on the efficacy of the Hib and pneumococcal conjugate (PCV) vaccines against pneumonia, as verified by X-ray, and pneumococcal and Hib disease. All observational and case–control Hib studies were excluded, due to the fact that the estimated efficacy of the vaccine was implausibly high. Furthermore, the Hib trial data only contained participants < 5 years old, and so Hib could not be modeled for those > 5 years old. As most PCV studies only included younger participants, to identify the contribution of pneumococcal pneumonia in older age groups, PCV efficacy studies using a before-after methodology were also included [15]. The self-reported prevalence of LRI symptoms, reported in the Demographic and Health Survey and the Multiple Indicator Cluster Survey, along with other population-representative surveys, were used to estimate the non-fatal burden of LRIs. Where possible, data were extracted from the survey data by sex and for each one-year age group. Furthermore, a formula was used to transform the bi-weekly prevalence into point prevalence data. There were four definitions used to measure LRI prevalence: (1) a cough with difficulty breathing, chest symptoms, and a fever, which was the gold standard definition (2) a cough, difficulty breathing and symptoms in the chest, but no fever, (3) a cough, difficulty breathing, and a fever, and (4) a cough without fever, but with difficulty breathing. The first definition was used as the reference definition; all other definitions were adjusted to this definition. LRI modeling included both inpatient and outpatient hospital data and the survey data was adjusted for seasonality. Prior to the modeling process, all data were adjusted to the reference definition, as well as for multiple admissions, multiple diagnoses, and outpatient claims. The data were first converted into prevalences, and were then compared to the LRI reference definition using a meta-regression model. Finally, the new data sources were added to (https://ghdx.healthdata.org/gbd-2019/data-input-sources) [15]. LRI mortality was estimated separately for males and females, for children under five and for those older than five, using a Cause of Death Ensemble model (CODEm). All available data from vital registration systems, surveillance systems, and verbal autopsies, were included in CODEm, with all outliers being carefully checked and excluded by country or region.

### Disease modeling

The non-fatal burden of LRIs was modelled using a Bayesian meta-regression modeling framework, Dis Mod-MR, which estimated the incidence, prevalence, and remission for each age, sex, geographic location, and year. The recovery period was on average 10 days (5–15 days), which relates to a remission period of 36.5 days. Country-level covariates were also included in the CODEm models [15]. Individual CODEm models were generated for those under 5 years old and those aged 5–95+ years old, due to differences in the patterns. All LRI mortality models were single-cause, which means that the sum of all mortality models will be the same as the all-cause mortality envelope. CoDCorrect was used to rescale the mortality estimates, in accordance with the uncertainty related to the specific mortality rate, in order to ensure internal consistency amongst the causes of death [15].

Two different counterfactual modeling strategies were used to estimate the population attributable fractions (PAFs) for the different etiologies, which have been reported previously [15]. The PAF shows the relative reduction in mortality if there was no exposure to an individual etiology. As LRIs can be caused by several different pathogens, which may also co-infect, PAFs may overlap and thus can equal more than 100%. The viral (influenza and RSV) and bacterial (streptococcus pneumonia and Hib) etiologies were estimated using different strategies. Unfortunately, due to a lack of quality data, the etiologies for neonatal deaths could not be attributed. The PAF uncertainty estimates were estimated using 1000 draws for each parameter, assuming log-normal distributions. A comprehensive account of the methodology used to calculate the PAFs, as well as the non-fatal and fatal burden of the different etiologies, have previously been reported [15].

### Compilation of results

All sequelae, and their associated disability weights (DWs), were obtained from the GBD 2013 European Disability Weights Measurement Study [15, 16]. A meta-analysis was used to establish the proportion in each severity category, moderate (85%) and severe (15%), which were then multiplied by the severity-specific DWs to generate the years lived with disability (YLDs) [15]. The number of deaths in an age group were multiplied by the remaining life expectancy in that age group, using the GBD standard life table, to produce the years of life lost (YLLs). The DALYs were estimated by summing the YLLs and YLDs. Uncertainty was estimated by sampling 1000 draws at each computational step, as well as including uncertainty from a number of sources (i.e., input data, measurement error, and estimates of residual non-sampling error). The uncertainty intervals (UIs) were specified as the 25th and 975th values of the ordered draws. Smoothing Splines models were used to investigate the relationship between the LRI burden (i.e., DALYs) and the SDI for the 21 countries located in the MENA region [15]. The SDI (Social Development Index), which is an estimate of development level, is a multifactorial measure that is comprised of income per capita, education level among the population aged 15+, and the total fertility rate among those < 25 years old. The SDI ranges from 0 (less developed) to 1 (most developed). The age-standardised annual incidence, deaths, and DALY rates were presented using R software (V 3.5.2). The average annual percent changes (AAPC) in the age-standardised incidence, death and DALYs attributable to LRIs in the 21 MENA countries were calculated using Joinpoint (v 4.9.1.0) software [17, 18]. Moreover, Pearson’s correlation coefficients were used to evaluate the relationship between the AAPCs and SDI using Stata software (v 14).

## Results

### The Middle East and North Africa region

In 2019, there were 3.42 million (95% UI: 3.17 million to 3.68 million) incident cases of LRIs in the MENA region, with an age-standardised rate of 6510.2 (6063.6–6997.8) per 100,000 population, which represents a 28.9% decrease since 1990 (− 30.8% to − 26.8%) (Table 1; Additional file 1: Table S1). LRIs accounted for 107,742 (94,479–122,048) deaths in 2019, with an age-standardised rate of 26.4 (23.2–29.6) per 100,000 population, which was 51.9% lower than in 1990 (− 59.2% to − 45.2%) (Table 1; Additional file 2: Table S2). In 2019, the number of regional DALYs was 4.72 million (3.99 million to 5.47 million), with an age-standardised rate of 888.5 (761.1–1019.9) DALYs per 100,000 population, a decrease of 71.4% since 1990 (− 77.8% to − 65.1%) (Table 1; Additional file 3: Table S3).

**Table 1:** Incident cases, deaths and DALYs for lower respiratory infection in 2019 and the percentage change in the age-standardised rates during the period 1990–2019

	Incidence (95% UI)			Deaths (95% UI)			DALYs (95% UI)		
	Counts (2019)	ASRs (2019)	Pcs in ASRs 1990–2019	Counts (2019)	ASRs (2019)	Pcs in ASRs 1990–2019	Counts (2019)	ASRs (2019)	Pcs in ASRs 1990–2019
North Africa and Middle East	34,197,034 (31,709,280, 36,805,894)	6510.2 6063.6, 6997.8)	− 28.9 (− 30.8, − 26.8)	107,742 (94,479, 122,048)	26.4 (23.2, 29.6)	− 51.9 (− 59.2, − 45.2)	4,716,300 (3,993,317, 5,473,257)	888.5 (761.1, 1019.9)	− 71.4 (− 77.8, − 65.1)
Afghanistan	2,546,222 (2,292,974, 2,842,980)	8037.7 (7400.8, 8709.2)	− 27.1 (− 31, − 23.2)	18,697 (14,418, 23,718)	61.9 (52.1, 72.6)	− 57.3 (− 68.4, − 46.3)	1,402,790 (1,028,351, 1,838,486)	2642.7 (2076.3, 3280.2)	− 71.5 (− 80, − 60.7)
Algeria	2,165,039 (1,997,696, 2,340,118)	5848.9 (5413.5, 6308.4)	− 27.2 (− 30.8, − 23.3)	5786 (4697, 7112)	23.3 (18.9, 29)	− 54.2 (− 64, − 43.6)	177,301 (145,455, 217,772)	505.6 (417.7, 618.5)	− 73.3 (− 82, − 62.8)
Bahrain	60,374 (54,753, 66,400)	5691.8 (5145.7, 6240.1)	− 19 (− 22.9, − 14.9)	83 (67, 101)	21.6 (16.4, 25.6)	− 28.4 (− 44.3, − 11.6)	2177 (1812, 2589)	321.9 (263, 379.6)	− 44.7 (− 55.7, − 32.2)
Egypt	6,998,178 (6,432,818, 7,588,299)	8150.8 (7535.8, 8783.5)	− 33.6 (− 37.4, − 29.5)	21,371 (16,332, 27,730)	33.7 (25.8, 43.9)	− 61.3 (− 70.7, − 48.9)	1,023,972 (755,472, 1,353,435)	1166.1 (884.6, 1523)	− 78.9 (− 85, − 70.8)
Iran (Islamic Republic of)	3,945,043 (3,660,046, 4,245,822)	5193 (4827.6, 5599)	− 34.1 (− 36.1, − 32)	10,219 (9150, 10,998)	16.4 (14.5, 17.8)	− 50.2 (− 57.6, − 43.8)	255,439 (232,950, 277,268)	359 (326.5, 390.5)	− 77 (− 82.8, − 71)
Iraq	1,971,734 (1,801,984, 2,166,866)	5533.1 (5103, 5988.9)	− 37.7 (− 41.2, − 33.4)	3178 (2605, 3866)	12.8 (10.7, 15.9)	− 63.6 (− 72.2, − 51.3)	159,655 (123,473, 204,684)	429.9 (342.4, 532.3)	− 78.6 (− 85.2, − 70.3)
Jordan	534,335 (483,138, 592,746)	5571.8 (5111.3, 6090.9)	− 31.7 (− 35.4, − 27.7)	1054 (878, 1275)	18.5 (15.5, 22)	− 44.3 (− 54.4, − 31.5)	47,065 (37,082, 60,195)	508.6 (412.8, 629.6)	− 56.6 (− 67.4, − 43.9)
Kuwait	211,005 (193,582, 228,655)	6583.2 (6041.4, 7204.4)	− 9.4 (− 13.9, − 4.6)	668 (551, 789)	35.7 (28.7, 42.6)	21.4 (1.2, 44.6)	14,477 (12,161, 17,029)	596.5 (502, 705.2)	− 21.4 (− 34.6, − 5.5)
Lebanon	285,941 (261,734, 313,270)	5578.4 (5103, 6122.1)	− 19.6 (− 23.6, − 15.1)	910 (737, 1293)	18.1 (14.7, 25.7)	− 35.4 (− 49.3, − 6.7)	18,592 (15,416, 23,512)	360.1 (298.7, 454.6)	− 55.9 (− 65.7, − 43.6)
Libya	340,992 (313,182, 368,631)	5983.2 (5508, 6470.7)	− 25.4 (− 29.3, − 21.4)	837 (665, 1038)	18.6 (14.8, 23.1)	− 30.8 (− 46.8, − 10.4)	22,863 (18,058, 28,781)	440 (349, 547.3)	− 55.9 (− 68.3, − 39.3)
Morocco	2,076,477 (1,920,514, 2,241,727)	6390 (5915.2, 6889.9)	− 29.4 (− 33, − 25.5)	6248 (4884, 7760)	24.8 (19.4, 30.9)	− 49.8 (− 59.9, − 38.3)	204,076 (148,367, 271,649)	686.5 (497.9, 917.8)	− 75.2 (− 82.9, − 65.2)
Oman	183,191 (166,881, 200,389)	6261.6 (5738, 6794.3)	− 33.3 (− 37.1, − 29.3)	407 (344, 466)	40.9 (32.8, 47.9)	− 28.5 (− 42.8, − 2.7)	12,839 (11,233, 14,564)	692.8 (578, 789.6)	− 52.8 (− 62.1, − 40.4)
Palestine	283,639 (256,822, 312,565)	6940.6 (6422, 7502.6)	− 35.9 (− 39.2, − 31.9)	500 (425, 646)	25.9 (21.7, 34.1)	− 31.8 (− 45.2, − 14.7)	16,501 (13,892, 20,029)	512.8 (436.6, 653.4)	− 52.8 (− 63.2, − 40.7)
Qatar	97,218 (86,992, 108,186)	5451.1 (4951.9, 5981.8)	− 19.8 (− 24, − 15)	70 (54, 93)	24.8 (20, 31)	− 8 (− 31.7, 19.3)	2726 (2111, 3636)	357.8 (288, 448.1)	− 38.2 (− 52.9, − 20.6)
Saudi Arabia	1,876,500 (1,710,127, 2,038,350)	7105.3 (6499.8, 7710.9)	− 28.8 (− 33, − 24.7)	4699 (3758, 5858)	32.2 (26.9, 38.9)	− 26.1 (− 42.5, 1.9)	156,031 (122,635, 199,355)	662.3 (541.1, 805.5)	− 38.5 (− 51.5, − 18.4)
Sudan	2,026,310 (1,841,827, 2,229,585)	6068.4 (5584.8, 6548.2)	− 34.4 (− 38, − 30.8)	7026 (5141, 9198)	29.4 (22.1, 37.2)	− 58.3 (− 72.5, − 43)	380,930 (256,106, 552,654)	951.9 (691.8, 1271.9)	− 76.1 (− 85.7, − 62.8)
Syrian Arab Republic	850,901 (784,572, 918,579)	6535.3 (6016.6, 7047.6)	− 18.9 (− 23, − 14.6)	2284 (1763, 2908)	23.9 (18.9, 30)	− 16.5 (− 37, 11)	79,826 (60,138, 102,141)	659.7 (507.6, 835.4)	− 52.4 (− 65.8, − 34.2)
Tunisia	687,208 (628,667, 745,739)	6097.2 (5586.3, 6630)	− 23.1 (− 27.8, − 18.7)	1838 (1388, 2424)	17.5 (13.2, 22.9)	− 48.7 (− 62.2, − 32.3)	41,698 (31,643, 54,752)	386.1 (294.2, 503.8)	− 75.8 (− 84.1, − 64.3)
Turkey	4,701,615 (4,354,663, 5,083,915)	5905.6 (5446.4, 6416.4)	− 25.7 (− 30.6, − 20.8)	14,868 (11,499, 17,816)	19 (14.8, 22.6)	− 63.6 (− 72.3, − 52.7)	309,439 (248,357, 367,147)	415.3 (339.1, 488.7)	− 87.5 (− 91.2, − 83.1)
United Arab Emirates	319,942 (288,108, 353,471)	6584.1 (6045, 7131.7)	− 19.7 (− 24.2, − 15.1)	599 (462, 760)	50.7 (27, 61.9)	− 39.3 (− 52.4, − 14.4)	19,275 (14,480, 26,407)	728.2 (496.9, 888.8)	− 44.7 (− 56.2, − 25.9)
Yemen	2,000,425 (1,822,340, 2,186,765)	8044.8 (7474.8, 8718.3)	− 31.7 (− 35.3, − 27.9)	6289 (4504, 8359)	35.8 (26, 49.7)	− 48.4 (− 66.1, − 27.5)	363,836 (243,983, 529,232)	1175 (834.6, 1563.3)	− 68.9 (− 80.5, − 53.3)

### National level

**Fig. 1:**
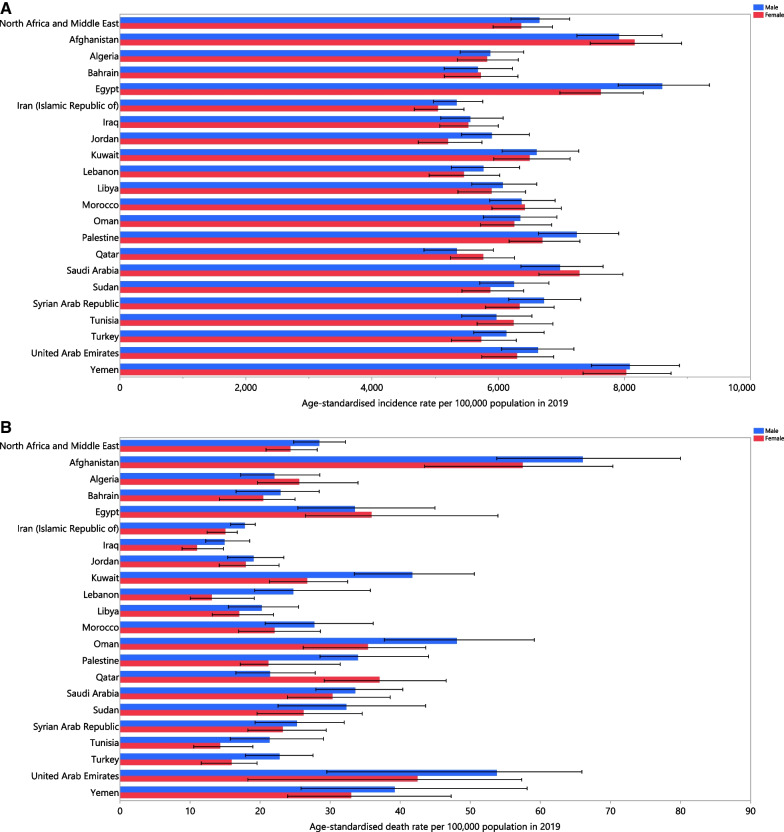

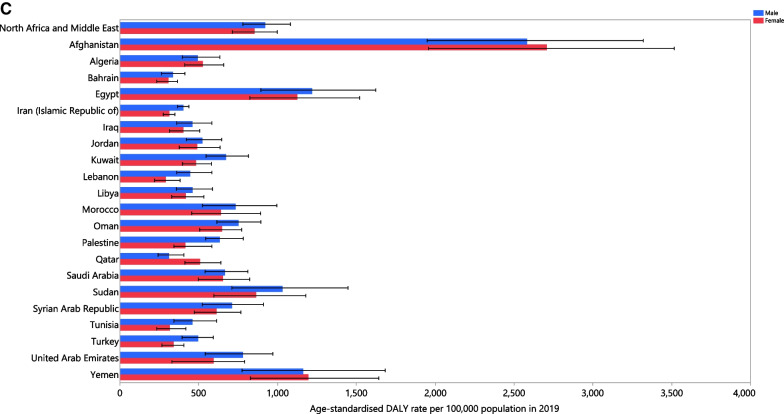
Age-standardised incidence (**A**), death (**B**), and DALYs (**C**) of lower respiratory infections (per 100,000 population) in the Middle East and North Africa region in 2019, by sex and country. DALY, disability-adjusted-life-years (generated from data available from http://ghdx.healthdata.org/gbd-results-tool)

In 2019, the age-standardised incidence rates of LRIs, among the countries that comprise the MENA region, ranged from 5193.0 to 8150.8 per 100,000 population. Egypt [8150.8 (7535.8–8783.5)], Yemen [8044.8 (7474.8–8718.3)] and Afghanistan [8037.7 (7400.8–8709.2)] had the three highest age-standardised incidence rates in 2019. In contrast, Iran [5193 (4827.6–5599)], Qatar [5451.1 (4951.9–5981.8)] and Iraq [5533.1 (5103–5988.9)] had the lowest age-standardised incidence rates (Fig. 1A; Additional file 1: Table S1).

In 2019, the national age-standardised death rates from LRIs ranged from 23.2 to 29.6 cases per 100,000 population in the MENA countries. The highest rates were observed in Afghanistan [61.9 (52.1–72.6)], the United Arab Emirates [50.7 (27.0–61.9)] and Oman [40.9 (32.8–47.9)], while the lowest rates were found in Iraq [12.8 (10.7–15.9)], Iran [16.4 (14.5–17.8)] and Tunisia [17.5 (13.2–22.9)] (Fig. 1B; Additional file 2: Table S2).

In 2019, the national age-standardised DALY rate of LRIs ranged from 761.1 to 1019.9 cases per 100,000 population. The highest rates were observed in Afghanistan [2642.7 (2076.3–3280.2)], Yemen [1175 (834.6–1563.3)] and Egypt [1166.1 (884.6–1523)]. Conversely, the lowest rates were seen in Bahrain [321.9 (263.0–379.6)], Qatar [357.8 (288.0–448.1)] and Iran [359.0 (326.5–390.5)] (Fig. 1C; Additional file 3: Table S3).

The percentage change in the age-standardised incidence rate decreased from 1990 to 2019 in all MENA countries, with Iraq [− 37.7% (− 41.2 to − 33.4%)], Palestine [− 35.9% (− 39.2 to − 31.9%)] and Sudan [− 34.4% (− 38.0 to − 30.8%)] having the largest decreases. In contrast, Kuwait [− 9.4% (− 13.9 to − 4.6%)] and the Syria [− 18.9% (− 23.0 to − 14.6%)] had the smallest decreases (Additional file 1: Table S1, Additional file 4: Figure S1).

There were no countries where the age-standardised death or DALY rates increased from 1990 to 2019. Iraq [− 63.6% (− 72.2 to − 51.3%)], Turkey [− 63.6% (− 72.3 to − 52.7%)] and Egypt [− 61.3% (− 70.7 to − 48.9%)] showed the largest decreases in the age-standardised death rates over the measurement period (Additional file 2: Table S2, Additional file 5: Figure S2). Similarly, Turkey [− 87.5% (− 91.2 to − 83.1%)], Egypt [− 78.9% (− 85.0 to − 70.8%)] and Iraq [− 78.6% (− 85.2 to − 70.3%)] showed the largest decreases in the age-standardised DALY rate over the same period (Additional file 3: Table S3, Additional file 6: Figure S3).

### Age and sex patterns

**Fig. 2:**
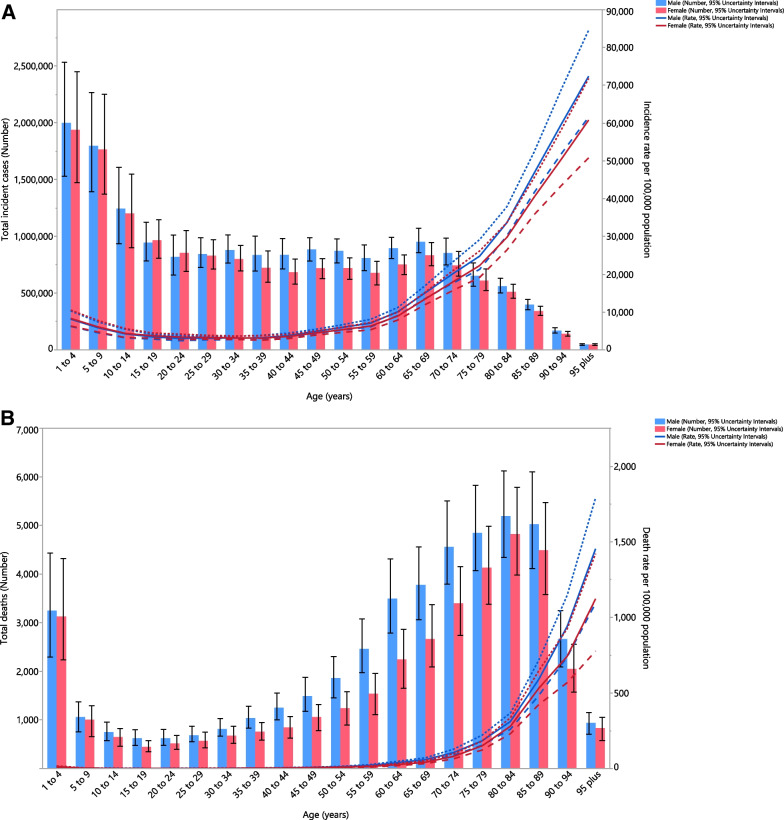

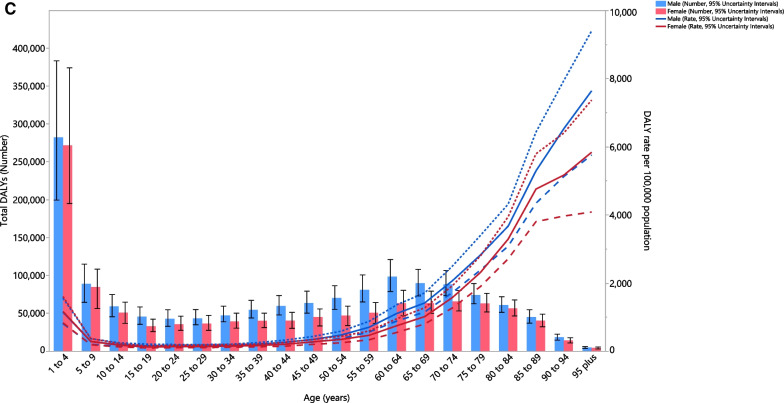
Numbers of incident cases and incidence rate (**A**), number of deaths and death rate (**B**) and the number of DALYs and DALY rate (**C**) for lower respiratory infections per 100,000 population in the Middle East and North Africa region, by age and sex in 2019; Dotted and dashed lines indicate 95% upper and lower uncertainty intervals, respectively. DALY, disability-adjusted-life-years (generated from data available from http://ghdx.healthdata.org/gbd-results-tool)

In 2019, the regional total incident cases of LRIs was highest in the 1–4 age groups in females and males. Moreover, the age-standardised incidence rates generally increased with advancing age in both sexes (Fig. 2A). In 2019, the regional total number of deaths due to LRIs was highest in the 80–84 age group among males and females. The age-standardised death rate was highest in the 95+ age group (Fig. 2B). The highest number of DALYs was in the 1–4 age group, while it was lowest in the 95+ age group in males and females. The regional age-standardised DALY rates for males and females steadily increased with advancing age, except for a decrease in the 5–9 age group (Fig. 2C).

**Fig. 3:**
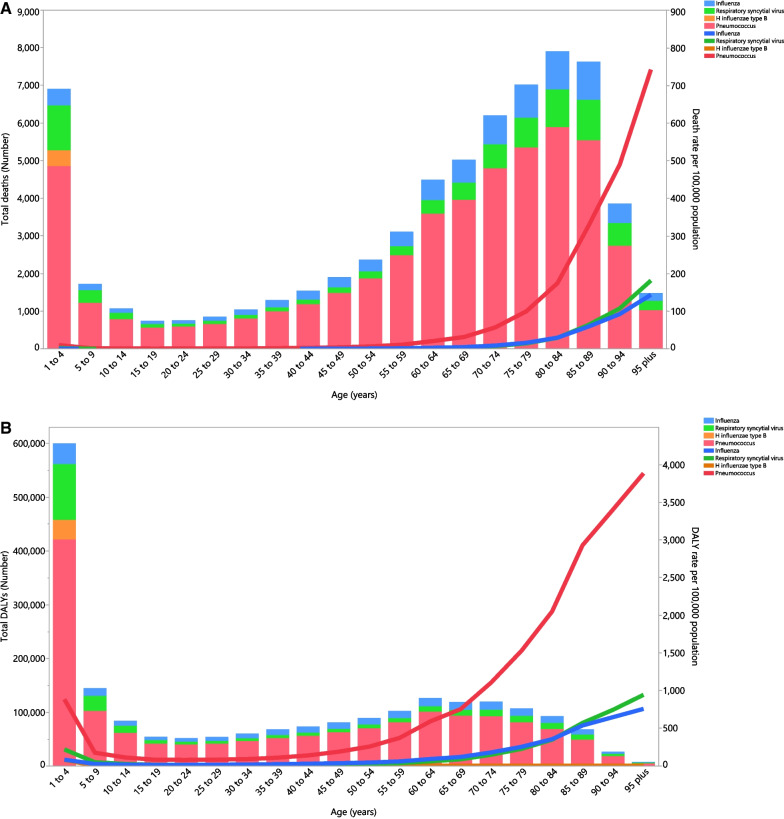
Numbers of deaths and death rate (**A**), and number of DALYs and DALY rate (**B**) for lower respiratory infections per 100,000 population in the Middle East and North Africa region per 100,000 population attributable to each underlying cause by age in 2019. The lines represent the death and DALY rates per 100,000 population in each age group. The bar charts represent the total number of deaths and DALYs in each age group (generated from data available from http://ghdx.healthdata.org/gbd-results-tool)

In 2019, pneumococcus and Hib were the most and least common etiology types, in terms of deaths (Fig. 3A). The largest number of deaths (per 100,000) from pneumococcus, influenza, RSV and Hib were found in the 80–84, 85–89, 1–4 and 1–4 age groups, respectively (Fig. 3A). The highest number of DALYs for pneumococcus, influenza, RSV and Hib were found in the 1–4 age group. The lowest number of DALYs from pneumococcus, influenza, RSV and Hib were found in the 95+ age group (Fig. 3B).

**Fig. 4:**
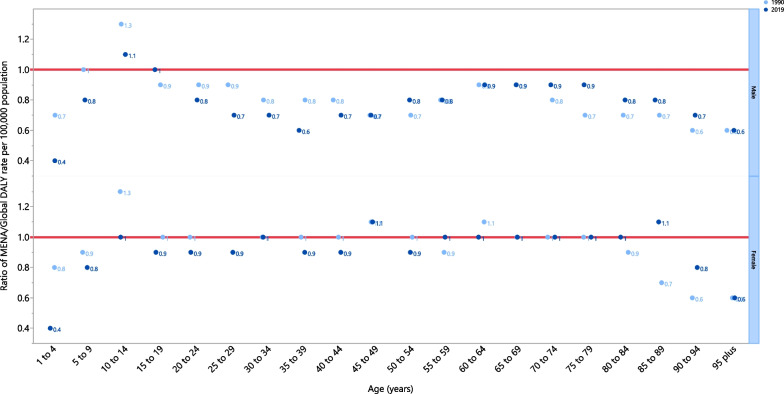
Ratio of the Middle East and North Africa region to the global lower respiratory infections DALY rate according to age group and sex, 1990–2019. DALY, disability-adjusted-life-years (generated from data available from http://ghdx.healthdata.org/gbd-results-tool)

The rate ratio, comparing the age-standardised DALY rates in MENA to the global rates for the different age groups, by sex in 1990 and 2019, showed that there were substantial variations from the reference values in males and females aged 1–4 and above 95 years old, while most other age groups were similar to the global average. However, females in the 45–49 age group had a higher LRI burden than the global average, for both 1990 and 2019. In contrast those in the 95+ age group, including both females and males, had a lower LRI burden than the global average for both 1990 and 2019 (Fig. 4).

### Association with the socio-demographic Index (SDI)

**Fig. 5:**
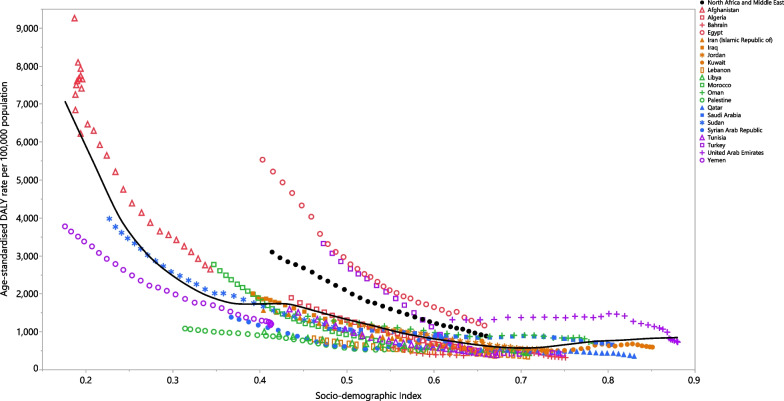
Age-standardised DALY rates of lower respiratory infections for 21 countries and territories, by SDI in 2019; expected values based on the Socio-demographic Index and disease rates in all locations are shown as the black line. Each point shows the observed age-standardised DALY rate for each country in 2019. DALY, disability-adjusted-life-years; SDI, socio-demographic Index (generated from data available from http://ghdx.healthdata.org/gbd-results-tool)

The burden of LRIs generally decreased, from 1990 to 2019, with increasing socio-economic development. Countries such as Egypt had much higher than expected burdens, whereas other countries had much lower than expected burdens (Fig. 5). There was a significant positive correlation between SDI and the AAPC for the incidence (ρ = 0.518; *p* = 0.013), deaths (ρ = 0.545; *p* = 0.009) and DALYs (ρ = 0.578; *p* = 0.005) in the MENA region.

### LRI joinpoint trends

Over the period 1990 to 2019, the regional incidences, deaths and DALYs attributable to LRIs decreased with AAPCs of − 1.19% (− 1.25 to − 1.13), − 2.47% (− 2.65 to − 2.28) and − 4.21% (− 4.43 to − 3.99), respectively (Additional file 1: Table S1, Additional file 2: Table S2 and Additional file 3: Table S3). The AAPCs for the LRI incidences decreased in all MENA countries from 1990 to 2019, with Iraq [− 1.64% (− 1.74 to − 1.53)] and Palestine [− 1.53% (− 1.63 to − 1.43)] having the largest decreases. In contrast, Kuwait [− 0.36% (− 0.42 to − 0.30)] and Bahrain [− 0.71% (− 0.77 to − 0.64)] had the smallest decreases (Additional file 1: Table S1). The AAPCs for LRI attributable deaths decreased in all countries, except for Kuwait [0.68% (− 0.46 to 1.84)]. The largest decreases were observed in Turkey [− 3.40% (− 3.87 to − 2.92)] and Iraq [− 3.39% (− 3.64 to − 3.14)], while Qatar [− 0.26% (− 2.61, 2.15) had the smallest decrease (Additional file 2: Table S2). The AAPCs in the LRI attributable DALYs decreased in all counties over the 1990–2019 period, with Turkey [− 6.89% (− 7.42 to − 6.30)] and Egypt [− 5.25% (− 5.53 to − 4.98)] having the largest decreases, while Kuwait [− 0.33% (− 1.11 to 0.47)] had the smallest decrease (Additional file 3: Table S3).

## Discussion

The present study showed that the burden attributable to LRIs decreased over the period 1990–2019 in all MENA countries. There were no substantial differences between males and females in the LRI burden. However, the highest numbers of incidence and DALYs were found in the 1–4 age group, whereas the highest death rate was among those aged 80–84. In terms of the underlying cause, pneumococcus accounted for the largest number of deaths and DALYs, and its rate increased with advancing age. Furthermore, countries with higher SDIs generally had lower age-standardised DALY rates attributable to LRIs and there was a significant positive correlation between SDI and the AAPC. A decreasing trend was observed for the age-standardised burden of LRIs, in terms of the AAPC.

A previous study conducted in the Eastern Mediterranean Region (EMR), using data from the GBD 2015 study, showed that the age-standardised DALY and death rates (per 100,000) were 1518 and 29.5, respectively [19]. Similarly, in 2019 we found the age-standardised DALY and death rates to be 888.5 and 26.4 per 100,000 in MENA, respectively. Moreover, the 2015 study revealed that the death and DALY rates decreased by 60.1% and 69.4% in the EMR between 1990 and 2015, respectively [19], while the present study found reductions of − 51.9% and − 71.4% in the age-standardised death and DALY rates, respectively, in MENA over the period 1990–2019. These relatively small differences could be as a result of the differences in the countries located in the EMR and MENA regions, as well as the fact that our study reported data from the most recent iteration of the GBD project. Another study, conducted in 2010 in several Arab countries, showed that the percent of DALYs was 6.0% in both sexes. The discrepancy in the attributable burden between that study and our own could be due to socio-demographic factors, maternal literacy, equal access to public health insurance and the prevention and treatment services, war, population growth, as well as differences in the age distribution in these countries [20]. As there were several disparities in the countries included in the previous research and their measurement periods were not the same, any inconsistencies are likely to be due to demographic differences.

In 2019, Iran and Iraq were the MENA countries with the lowest age-standardised incidence and death rates. Moradi-Lakeh et al. reported that in 2015 Lebanon and Qatar had the lowest age-standardised death and DALY rates in the EMR, while the lowest age-standardised incidence rate was found in the UAE [19]. These different findings may be due to the implementation of interventions and programs which have been conducted in some of the MENA countries, as well as due to reductions in exposure to potential risk factors. For example, reductions in several LRI risk factors occurred over the period 2005–2015, such as childhood wasting, household air pollution from solid fuels, second-hand smoke, child stunting, child underweight, non-exclusive breast feeding, and zinc deficiency, which would have contributed to the reduction in the LRI-related deaths and DALYs in MENA [21]. Moreover, vaccine coverage for Hib and pneumococcus, which are common etiologic factors for LRIs, increased over the period 2010–2019 [22]. The Global Vaccine Action Plan (GVAP) aimed to reach at least 90% vaccine coverage for all assessed vaccines in each country by 2020 [23]. However, by 2019 only 11 countries and territories across the world had reached the target for all assessed vaccines [22]. In MENA, the proportion of countries which reached the target increased from zero in 1990 to 24% in 2019 [22]. Furthermore, the proportion of locations in MENA that reached the GVAP target for the third does of the Haemophilus influenzae type b vaccine increased from 52% in 2010 to 62% in 2019, while the third dose of the pneumococcal conjugate vaccine also increased from 33 to 52% during that period [22]. It seems that the GVAP target could not be achievable for all countries in the MENA region, particularly in countries that have high incidence rates and the lowest decreases in the adjusted incidence rates. Minority religions, a lack of understanding about the importance of vaccinations and the COVID-19 pandemic may have prevented some countries from achieving the GVAP target [24, 25]. Conversely, ambient particulate matter pollution and poor availability of antibiotics increased in MENA between 2000 and 2016, and health policymakers must take steps to resolve these problems [26]. Moreover, regional health authorities and other health-related organizations should develop plans to enable more MENA countries to reach the GVAP target in the next few years.

The present study found that most attributable DALYs and deaths were among children under 5 years old and those more than 80 years of age. The largest etiological causes of LRIs in MENA were pneumococcus, followed by RSV and influenza. In accordance with our report, using data from GBD 2016, Troeger and colleagues showed that in 2016 pneumococcus and RSV were responsible for 50.1% and 3.2% of the attributable deaths among all ages, respectively [27]. In children younger than 5 years old and adults above 70 years, the second and third most common causes were Hib (7.4%) and influenza (2.3%), respectively [27].

There are several strategies which can be used to reduce the attributable burden and death due to LRIs in children. Firstly, developing an easy-to-access diagnostic test with high sensitivity and specificity will help physicians to accurately diagnose LRIs, which could also lead to a decrease in the inappropriate use of antibiotics and thereby a reduction in drug-resistant bacteria [28]. Also, providing specific tests to reduce inappropriate antibiotic use and thereby drug resistance are recommended. Secondly, the results of a meta-analysis revealed that routine zinc supplementation in young children can be used as a prophylactic measure to reduce the incidence of acute LRIs by around 35% in developing countries [29]. Thirdly, the substantial preventative role of pneumococcal vaccinations for children, and even adults, should be highlighted and programs should be developed to encourage individuals to participate in vaccination programmes [30]. Fourthly, the reduction of RSV modifiable risk factors in children should be undertaken, such as not being breastfed, human immunodeficiency virus infection, maternal smoking, and household crowding. In addition, resources must be allocated for the development of RSV vaccines and monoclonal antibodies to reduce the RSV-attributable burden of LRIs [31]. Fifthly, because micronutrients like vitamin A, iron, folic acid, vitamin D, calcium, as well as breastfeeding help to prevent LRIs, multicomponent nutritional intervention programs are needed. These programs must be initiated prior to pregnancy and should be a priority for countries with high-levels of LRI attributable incidence and deaths, such as Egypt, Yemen and Afghanistan [32]. Moreover, further studies on the strategies to reduce and control the burden attributable to LRIs are highly recommended, especially in the MENA region.

The age-standardised incidence, death and DALY rates increased with advancing age, with those over 95 years of age having the highest rates. The results of a prospective observational study on 587 participants aged 85 and above showed that smoking, severe cognitive impairment, a history of stroke and impaired functional status, oral glucocorticoid use and a history of chronic obstructive pulmonary disease (COPD) were associated with LRIs [33]. Therefore, measures such as the implementation of tobacco smoking cessation programs, improving the social situation and psychological state of the elderly, the prevention or control of chronic diseases (e.g., stroke and COPD) should be implemented to help reduce the burden and deaths among the elderly affected by LRIs [34]. Moreover, because influenza is the second largest contributor to the LRI burden, after pneumococcus, the prevention of influenza by annual vaccination should be undertaken in populations at a higher risk of medical complications, such as children aged 6–59 months, adults aged 50 and older, individuals with chronic pulmonary, cardiovascular, renal, neurologic, hepatic, hematologic or metabolic disorders, and residents of nursing homes [35]. The present report did not find large differences in the LRI burden between males and females. In accordance with our findings, the GBD 2015 data also showed no substantial differences between males and females in the age-standardised incidence of LRIs in the EMR, although it was slightly higher in females in all countries except Pakistan [19]. Furthermore, a similar sex pattern was found in 2015 for the burden of LRIs at the global level [12].

The present study revealed that there was a negative association between SDI and the LRI-attributable burden. In accordance with our findings, at global and regional levels, previous studies have found a negative association between LRI-related fatalities and SDI in 2016 [27], and between SDI and the LRI-related incidence and mortality rates in 2015 [12]. Additionally, we found SDI had significant positive correlations with the AAPC for the incidence, death and DALYs. These findings also highlight the fact that there is a need to provide programmes for the future prevention of LRIs, particularly in countries with high socioeconomic development.

### Strengths and limitations of the study

The present study provides the most up-to-date information on the burden of LRIs and their underlying etiologies at the regional and national levels. A consistent and reproducible approach was used in GBD 2019 to estimate the burden of LRIs due to influenza and RSV. Furthermore, in previous GBD iterations the odds ratios for influenza and RSV were based upon a single study among children younger than five years old. However, with the recently published article on the odds of these pathogens in adults over 65 years, IHME was able to have different values by age and to produced more accurate LRI estimates by underlying etiology. Nevertheless, we acknowledge that the present report also has some limitations. Firstly, data was sparse in some locations, which will affect the interpretation of our results. Secondly, a clinical diagnosis of LRIs was included in this study without any further laboratory or imaging assessment, which might result in excessive variation and recall bias. Thirdly, GBD only reported the severity of LRIs that were associated with mortality and morbidity, although the effects of severity were adjusted in the models. Fourthly, variations in vaccine coverage and treatment utilization could interfere with the estimated LRI burden. In order to reduce this issue, the model was adjusted to account for vaccine coverage and the expected vaccine performance. Finally, the burden of LRIs attributable to each risk factors were not reported in the present manuscript, but they should be included in future studies.

## Conclusions

The LRI-associated burden in MENA decreased between 1990 and 2019. However, LRIs are responsible for a large number of incidence and deaths. Furthermore, we found that SDI had a significant positive correlation with the AAPC, and pneumococcus was the most common underlying cause of LRIs. Nevertheless, further studies are needed to assess the effectiveness of healthcare interventions and programs to control LRIs and their risk factors.

## Supplementary Information

**Additional file 1.** **Table S1**: Incidence of lower respiratory infections in 1990 and 2019 for both sexes and percentage change in age-standardised rates (ASRs) per 100000 in the North Africa and the Middle East region (Generated from data available from http://ghdx.healthdata.org/gbd-results-tool).

**Additional file 2.** **Table S2**: Deaths of lower respiratory infections in 1990 and 2019 for both sexes and percentage change in age-standardised rates (ASRs) per 100000 in the North Africa and the Middle East region (Generated from data available from http://ghdx.healthdata.org/gbd-results-tool).

**Additional file 3.** **Table S3**: DALYs due to lower respiratory infections in 1990 and 2019 for both sexes and percentage change in age-standardised rates (ASRs) per 100000 in the North Africa and the Middle East region. DALY= disability-adjusted-life-years. (Generated from data available from http://ghdx.healthdata.org/gbd-resultstool).

**Additional file 4.** **Figure S1**: The percentage change in the age-standardised incidence of lower respiratory infections in the Middle East and North Africa region from 1990 to 2019, by sex and country. (Generated from data available from http://ghdx.healthdata.org/gbd-results-tool).

**Additional file 5.** **Figure S2**: The percentage change in the age-standardised death of lower respiratory infections in the Middle East and North Africa region from 1990 to 2019, by sex and country. (Generated from data available from http://ghdx.healthdata.org/gbd-results-tool).

**Additional file 6.** **Figure S3**: The percentage change in the age-standardised DALYs of lower respiratory infections in the Middle East and North Africa region from 1990 to 2019, by sex and country. DALY= disability-adjusted-life-years. (Generated from data available from http://ghdx.healthdata.org/gbd-results-tool).

## Data Availability

The data used for these analyses are all publicly available at http://ghdx.healthdata.org/gbd-results-tool. This study is based on publicly available data and solely reflects the opinions of its authors and not that of the Institute for Health Metrics and Evaluation.
