# Supplementary material for: The burden of lower respiratory infections and their underlying etiologies in the Middle East and North Africa region, 1990–2019: results from the Global Burden of Disease Study 2019
Source: BMC Pulm Med. 2023 Jan 4;23:2. doi: 10.1186/s12890-022-02301-7 (PMC9811697; doi:10.1186/s12890-022-02301-7)
Supplement: Supplementary file 1 — Additional file 1. Table S1: Incidence of lower respiratory infections in 1990 and 2019 for both sexes and percentage change in age-standardised rates (ASRs) per 100000 in the North Africa and the Middle East region (Generated from data available from http://ghdx.healthdata.org/gbd-results-tool). [file 12890_2022_2301_MOESM1_ESM.docx]

| **Table S1: Incidence of lower respiratory infections in 1990 and 2019 and the percentage change in the age-standardised rates (ASRs) per 100,000 in the North Africa and the Middle East region**  **(Generated from data available from http://ghdx.healthdata.org/gbd-results-tool)** | | | | | | |
| --- | --- | --- | --- | --- | --- | --- |
|  | **1990** | | **2019** | | **Percentage change in ASRs per 100,000** | **Average annual % change**  **1990-2019** |
|  | **No (95% UI)** | **ASRs per 100,000 (95% UI)** | **No (95% UI)** | **ASRs per 100,000 (95% UI)** |  |  |
| **North Africa and Middle East** | **30314474 (27395800 , 33634682)** | **9152.7 (8478 , 9875.2)** | **34197034 (31709280 , 36805894)** | **6510.2 (6063.6 , 6997.8)** | **-28.9 (-30.8 , -26.8)** | **-1.19**  **(-1.25, -1.13)** |
| **Afghanistan** | **1311680 (1167335 , 1475599)** | **11025.2 (10044 , 12043.3)** | **2546222 (2292974 , 2842980)** | **8037.7 (7400.8 , 8709.2)** | **-27.1 (-31 , -23.2)** | **-1.11**  **(-1.18, -1.03)** |
| **Algeria** | **1828334 (1659833 , 2026974)** | **8032 (7409.5 , 8671)** | **2165039 (1997696 , 2340118)** | **5848.9 (5413.5 , 6308.4)** | **-27.2 (-30.8 , -23.3)** | **-1.08**  **(-1.18, -0.99)** |
| **Bahrain** | **27377 (23866 , 31105)** | **7025 (6318.2 , 7734.1)** | **60374 (54753 , 66400)** | **5691.8 (5145.7 , 6240.1)** | **-19 (-22.9 , -14.9)** | **-0.71**  **(-0.77, -0.64)** |
| **Egypt** | **6886857 (6191167 , 7672720)** | **12276.1 (11297.1 , 13351.4)** | **6998178 (6432818 , 7588299)** | **8150.8 (7535.8 , 8783.5)** | **-33.6 (-37.4 , -29.5)** | **-1.42**  **(-1.49, -1.35)** |
| **Iran (Islamic Republic of)** | **4176991 (3769247 , 4629194)** | **7877.7 (7322.9 , 8489.8)** | **3945043 (3660046 , 4245822)** | **5193 (4827.6 , 5599)** | **-34.1 (-36.1 , -32)** | **-1.45**  **(-1.53, -1.37)** |
| **Iraq** | **1593080 (1416257 , 1804018)** | **8878.5 (8122.5 , 9746.4)** | **1971734 (1801984 , 2166866)** | **5533.1 (5103 , 5988.9)** | **-37.7 (-41.2 , -33.4)** | **-1.64**  **(-1.74, -1.53)** |
| **Jordan** | **259966 (233475 , 291468)** | **8161 (7540.1 , 8829.6)** | **534335 (483138 , 592746)** | **5571.8 (5111.3 , 6090.9)** | **-31.7 (-35.4 , -27.7)** | **-1.29**  **(-1.37, -1.21)** |
| **Kuwait** | **97420 (87288 , 108853)** | **7268.2 (6687.6 , 7916.5)** | **211005 (193582 , 228655)** | **6583.2 (6041.4 , 7204.4)** | **-9.4 (-13.9 , -4.6)** | **-0.36**  **(-0.42, -0.30)** |
| **Lebanon** | **215206 (193820 , 236683)** | **6939.8 (6373.8 , 7526.1)** | **285941 (261734 , 313270)** | **5578.4 (5103 , 6122.1)** | **-19.6 (-23.6 , -15.1)** | **-0.75**  **(-0.82, -0.69)** |
| **Libya** | **311803 (278717 , 349293)** | **8019.8 (7332.7 , 8723.7)** | **340992 (313182 , 368631)** | **5983.2 (5508 , 6470.7)** | **-25.4 (-29.3 , -21.4)** | **-1.00**  **(-1.14, -0.85)** |
| **Morocco** | **2175758 (1968273 , 2404930)** | **9051.7 (8317.6 , 9805.9)** | **2076477 (1920514 , 2241727)** | **6390 (5915.2 , 6889.9)** | **-29.4 (-33 , -25.5)** | **-1.22**  **(-1.30, -1.14)** |
| **Oman** | **148099 (132084 , 167490)** | **9391.8 (8601.9 , 10218.9)** | **183191 (166881 , 200389)** | **6261.6 (5738 , 6794.3)** | **-33.3 (-37.1 , -29.3)** | **-1.39**  **(-1.45, -1.34)** |
| **Palestine** | **222731 (200242 , 249012)** | **10827.5 (10019.9 , 11692.6)** | **283639 (256822 , 312565)** | **6940.6 (6422 , 7502.6)** | **-35.9 (-39.2 , -31.9)** | **-1.53**  **(-1.63, -1.43)** |
| **Qatar** | **21976 (19709 , 24550)** | **6792.8 (6211.8 , 7389.7)** | **97218 (86992 , 108186)** | **5451.1 (4951.9 , 5981.8)** | **-19.8 (-24 , -15)** | **-0.78**  **(-0.83, -0.72)** |
| **Saudi Arabia** | **1291892 (1164840 , 1437455)** | **9985.5 (9195.2 , 10796.9)** | **1876500 (1710127 , 2038350)** | **7105.3 (6499.8 , 7710.9)** | **-28.8 (-33 , -24.7)** | **-1.17**  **(-1.23, -1.12)** |
| **Sudan** | **1869756 (1671042 , 2119517)** | **9254.6 (8502.6 , 10070.5)** | **2026310 (1841827 , 2229585)** | **6068.4 (5584.8 , 6548.2)** | **-34.4 (-38 , -30.8)** | **-1.46**  **(-1.52, -1.40)** |
| **Syrian Arab Republic** | **1059413 (941304 , 1195988)** | **8055.4 (7402.5 , 8758.6)** | **850901 (784572 , 918579)** | **6535.3 (6016.6 , 7047.6)** | **-18.9 (-23 , -14.6)** | **-0.72**  **(-0.85, -0.59)** |
| **Tunisia** | **617594 (559515 , 684461)** | **7931.3 (7310.6 , 8629.5)** | **687208 (628667 , 745739)** | **6097.2 (5586.3 , 6630)** | **-23.1 (-27.8 , -18.7)** | **-0.90**  **(-1.03, -0.77)** |
| **Turkey** | **4436875 (4005030 , 4936842)** | **7949.7 (7278.5 , 8662.2)** | **4701615 (4354663 , 5083915)** | **5905.6 (5446.4 , 6416.4)** | **-25.7 (-30.6 , -20.8)** | **-1.00**  **(-1.18, -0.82)** |
| **United Arab Emirates** | **89826 (80250 , 100655)** | **8203.1 (7545.1 , 8902.6)** | **319942 (288108 , 353471)** | **6584.1 (6045 , 7131.7)** | **-19.7 (-24.2 , -15.1)** | **-0.76**  **(-0.76, -0.82)** |
| **Yemen** | **1651451 (1456473 , 1879830)** | **11786.1 (10816.9 , 12821.9)** | **2000425 (1822340 , 2186765)** | **8044.8 (7474.8 , 8718.3)** | **-31.7 (-35.3 , -27.9)** | **-1.32**  **(-1.39, -1.26)** |
