# Supplementary material for: The burden of lower respiratory infections and their underlying etiologies in the Middle East and North Africa region, 1990–2019: results from the Global Burden of Disease Study 2019
Source: BMC Pulm Med. 2023 Jan 4;23:2. doi: 10.1186/s12890-022-02301-7 (PMC9811697; doi:10.1186/s12890-022-02301-7)
Supplement: Supplementary file 2 — Additional file 2. Table S2: Deaths of lower respiratory infections in 1990 and 2019 for both sexes and percentage change in age-standardised rates (ASRs) per 100000 in the North Africa and the Middle East region (Generated from data available from http://ghdx.healthdata.org/gbd-results-tool). [file 12890_2022_2301_MOESM2_ESM.docx]

| **Table S2: Deaths from lower respiratory infections in 1990 and 2019 and the percentage change in the age-standardised rates (ASRs) per 100,000 in the North Africa and the Middle East region**  **(Generated from data available from http://ghdx.healthdata.org/gbd-results-tool)** | | | | | | |
| --- | --- | --- | --- | --- | --- | --- |
|  | **1990** | | **2019** | | **Percentage change in ASRs per 100,000** | **Average annual % change**  **1990-2019** |
|  | **No (95% UI)** | **ASRs per 100,000 (95% UI)** | **No (95% UI)** | **ASRs per 100,000 (95% UI)** |  |  |
| **North Africa and Middle East** | **197703 (168472 , 245401)** | **54.9 (49.2 , 62.5)** | **107742 (94479 , 122048)** | **26.4 (23.2 , 29.6)** | **-51.9 (-59.2 , -45.2)** | **-2.47**  **(-2.65, -2.28)** |
| **Afghanistan** | **23741 (17931 , 32333)** | **145.2 (115.4 , 183.3)** | **18697 (14418 , 23718)** | **61.9 (52.1 , 72.6)** | **-57.3 (-68.4 , -46.3)** | **-2.87**  **(-3.27, -2.46)** |
| **Algeria** | **8125 (6021 , 11240)** | **50.8 (41.1 , 61.5)** | **5786 (4697 , 7112)** | **23.3 (18.9 , 29)** | **-54.2 (-64 , -43.6)** | **-2.66**  **(-2.77, -2.56)** |
| **Bahrain** | **38 (33 , 43)** | **30.2 (26 , 34.5)** | **83 (67 , 101)** | **21.6 (16.4 , 25.6)** | **-28.4 (-44.3 , -11.6)** | **-1.18**  **(-2.15, -0.20)** |
| **Egypt** | **56449 (49213 , 65790)** | **86.9 (78 , 97.8)** | **21371 (16332 , 27730)** | **33.7 (25.8 , 43.9)** | **-61.3 (-70.7 , -48.9)** | **-3.20**  **(-3.36, -3.04)** |
| **Iran (Islamic Republic of)** | **15595 (12752 , 20079)** | **33 (29.4 , 37.7)** | **10219 (9150 , 10998)** | **16.4 (14.5 , 17.8)** | **-50.2 (-57.6 , -43.8)** | **-2.42**  **(-2.50, -2.35)** |
| **Iraq** | **7337 (5768 , 9459)** | **35.1 (29.4 , 41.9)** | **3178 (2605 , 3866)** | **12.8 (10.7 , 15.9)** | **-63.6 (-72.2 , -51.3)** | **-3.39**  **(-3.64, -3.14)** |
| **Jordan** | **780 (643 , 955)** | **33.3 (28.4 , 38.7)** | **1054 (878 , 1275)** | **18.5 (15.5 , 22)** | **-44.3 (-54.4 , -31.5)** | **-1.96**  **(-2.40, -1.51)** |
| **Kuwait** | **191 (175 , 209)** | **29.4 (25.4 , 32.3)** | **668 (551 , 789)** | **35.7 (28.7 , 42.6)** | **21.4 (1.2 , 44.6)** | **0.68**  **(-0.46, 1.84)** |
| **Lebanon** | **647 (543 , 765)** | **28 (24 , 33)** | **910 (737 , 1293)** | **18.1 (14.7 , 25.7)** | **-35.4 (-49.3 , -6.7)** | **-1.49**  **(-1.59, -1.40)** |
| **Libya** | **840 (660 , 1068)** | **26.9 (21.8 , 32.8)** | **837 (665 , 1038)** | **18.6 (14.8 , 23.1)** | **-30.8 (-46.8 , -10.4)** | **-1.23**  **(-1.62, -0.85)** |
| **Morocco** | **12330 (9986 , 15047)** | **49.5 (41.2 , 58.5)** | **6248 (4884 , 7760)** | **24.8 (19.4 , 30.9)** | **-49.8 (-59.9 , -38.3)** | **-2.37**  **(-2.68, -2.06)** |
| **Oman** | **438 (345 , 545)** | **57.2 (44.4 , 69.4)** | **407 (344 , 466)** | **40.9 (32.8 , 47.9)** | **-28.5 (-42.8 , -2.7)** | **-1.14**  **(-1.58, -0.70)** |
| **Palestine** | **487 (384 , 606)** | **38 (30.4 , 51)** | **500 (425 , 646)** | **25.9 (21.7 , 34.1)** | **-31.8 (-45.2 , -14.7)** | **-1.28**  **(-1.42, -1.13)** |
| **Qatar** | **26 (21 , 34)** | **27 (21.9 , 37.1)** | **70 (54 , 93)** | **24.8 (20 , 31)** | **-8 (-31.7 , 19.3)** | **-0.26**  **(-2.61, 2.15)** |
| **Saudi Arabia** | **2812 (2242 , 3445)** | **43.6 (33.9 , 54.3)** | **4699 (3758 , 5858)** | **32.2 (26.9 , 38.9)** | **-26.1 (-42.5 , 1.9)** | **-1.07**  **(-1.14, -0.99)** |
| **Sudan** | **17665 (10866 , 29396)** | **70.5 (51.8 , 98.4)** | **7026 (5141 , 9198)** | **29.4 (22.1 , 37.2)** | **-58.3 (-72.5 , -43)** | **-2.98**  **(-3.11, -2.85)** |
| **Syrian Arab Republic** | **3592 (2780 , 4713)** | **28.6 (23.8 , 35.3)** | **2284 (1763 , 2908)** | **23.9 (18.9 , 30)** | **-16.5 (-37 , 11)** | **-0.70**  **(-1.26, -0.14)** |
| **Tunisia** | **2348 (1858 , 2975)** | **34.1 (29 , 40.5)** | **1838 (1388 , 2424)** | **17.5 (13.2 , 22.9)** | **-48.7 (-62.2 , -32.3)** | **-2.32**  **(-2.42, -2.22)** |
| **Turkey** | **32062 (24608 , 42458)** | **52.3 (42.9 , 65.5)** | **14868 (11499 , 17816)** | **19 (14.8 , 22.6)** | **-63.6 (-72.3 , -52.7)** | **-3.40**  **(-3.87, -2.92)** |
| **United Arab Emirates** | **177 (144 , 212)** | **83.6 (42 , 100)** | **599 (462 , 760)** | **50.7 (27 , 61.9)** | **-39.3 (-52.4 , -14.4)** | **-1.65**  **(-2.35, -0.94)** |
| **Yemen** | **11890 (7907 , 19469)** | **69.4 (52.1 , 90.9)** | **6289 (4504 , 8359)** | **35.8 (26 , 49.7)** | **-48.4 (-66.1 , -27.5)** | **-2.26**  **(-2.38, -2.14)** |
