# Supplementary material for: The burden of lower respiratory infections and their underlying etiologies in the Middle East and North Africa region, 1990–2019: results from the Global Burden of Disease Study 2019
Source: BMC Pulm Med. 2023 Jan 4;23:2. doi: 10.1186/s12890-022-02301-7 (PMC9811697; doi:10.1186/s12890-022-02301-7)
Supplement: Supplementary file 3 — Additional file 3. Table S3: DALYs due to lower respiratory infections in 1990 and 2019 for both sexes and percentage change in age-standardised rates (ASRs) per 100000 in the North Africa and the Middle East region. DALY= disability-adjusted-life-years. (Generated from data available from http://ghdx.healthdata.org/gbd-resultstool). [file 12890_2022_2301_MOESM3_ESM.docx]

| **Table S3: DALYs due to lower respiratory infections in 1990 and 2019 and the percentage change in the age-standardised rates (ASRs) per 100,000 in the North Africa and the Middle East region**  **(Generated from data available from http://ghdx.healthdata.org/gbd-results-tool)** | | | | | | |
| --- | --- | --- | --- | --- | --- | --- |
|  | **1990** | | **2019** | | **Percentage change in ASRs per 100,000** | **Average annual % change**  **1990-2019** |
|  | **No (95% UI)** | **ASRs per 100,000 (95% UI)** | **No (95% UI)** | **ASRs per 100,000 (95% UI)** |  |  |
| **North Africa and Middle East** | **15176430 (12652761 , 19535942)** | **3101.8 (2636.4 , 3870.7)** | **4716300 (3993317 , 5473257)** | **888.5 (761.1 , 1019.9)** | **-71.4 (-77.8 , -65.1)** | **-4.21**  **(-4.43, -3.99)** |
| **Afghanistan** | **1898139 (1407505 , 2647037)** | **9261.1 (6995.7 , 12599.5)** | **1402790 (1028351 , 1838486)** | **2642.7 (2076.3 , 3280.2)** | **-71.5 (-80 , -60.7)** | **-4.18**  **(-4.96, -3.40)** |
| **Algeria** | **555621 (369541 , 827957)** | **1897 (1393.6 , 2644.1)** | **177301 (145455 , 217772)** | **505.6 (417.7 , 618.5)** | **-73.3 (-82 , -62.8)** | **-4.45**  **(-4.59, -4.32)** |
| **Bahrain** | **1662 (1388 , 2011)** | **582.5 (509.2 , 660)** | **2177 (1812 , 2589)** | **321.9 (263 , 379.6)** | **-44.7 (-55.7 , -32.2)** | **-2.05**  **(-2.70, -1.39)** |
| **Egypt** | **4477548 (3814309 , 5302156)** | **5535.6 (4817.8 , 6467)** | **1023972 (755472 , 1353435)** | **1166.1 (884.6 , 1523)** | **-78.9 (-85 , -70.8)** | **-5.25**  **(-5.53, -4.98)** |
| **Iran (Islamic Republic of)** | **1161357 (912538 , 1571576)** | **1563.8 (1280.6 , 2009.3)** | **255439 (232950 , 277268)** | **359 (326.5 , 390.5)** | **-77 (-82.8 , -71)** | **-5.01**  **(-5.28, -4.75)** |
| **Iraq** | **572297 (439327 , 756419)** | **2007.8 (1573.9 , 2578.4)** | **159655 (123473 , 204684)** | **429.9 (342.4 , 532.3)** | **-78.6 (-85.2 , -70.3)** | **-5.14**  **(-5.41, -4.87)** |
| **Jordan** | **53643 (41703 , 68430)** | **1172.3 (979.2 , 1417.3)** | **47065 (37082 , 60195)** | **508.6 (412.8 , 629.6)** | **-56.6 (-67.4 , -43.9)** | **-2.84**  **(-3.04, -2.60)** |
| **Kuwait** | **9951 (8776 , 11429)** | **758.7 (690.7 , 832.5)** | **14477 (12161 , 17029)** | **596.5 (502 , 705.2)** | **-21.4 (-34.6 , -5.5)** | **-0.33**  **(-1.11, 0.47)** |
| **Lebanon** | **29841 (23393 , 37990)** | **816 (671.7 , 984.4)** | **18592 (15416 , 23512)** | **360.1 (298.7 , 454.6)** | **-55.9 (-65.7 , -43.6)** | **-2.77**  **(-2.92,-2.63)** |
| **Libya** | **52540 (38277 , 71721)** | **998.1 (783.7 , 1279.2)** | **22863 (18058 , 28781)** | **440 (349 , 547.3)** | **-55.9 (-68.3 , -39.3)** | **-2.75**  **(-3.07, -2.44)** |
| **Morocco** | **929403 (728952 , 1163357)** | **2773.3 (2225 , 3417.2)** | **204076 (148367 , 271649)** | **686.5 (497.9 , 917.8)** | **-75.2 (-82.9 , -65.2)** | **-4.67**  **(-4.98, -4.36)** |
| **Oman** | **24077 (17932 , 32730)** | **1466.5 (1168.2 , 1799.6)** | **12839 (11233 , 14564)** | **692.8 (578 , 789.6)** | **-52.8 (-62.1 , -40.4)** | **-2.53**  **(-2.99, -2.05)** |
| **Palestine** | **27414 (20309 , 37375)** | **1086 (861.8 , 1350.7)** | **16501 (13892 , 20029)** | **512.8 (436.6 , 653.4)** | **-52.8 (-63.2 , -40.7)** | **-2.57**  **(-2.79, -2.34)** |
| **Qatar** | **1440 (1090 , 1884)** | **579 (467.6 , 764)** | **2726 (2111 , 3636)** | **357.8 (288 , 448.1)** | **-38.2 (-52.9 , -20.6)** | **-1.75**  **(-2.88, -0.66)** |
| **Saudi Arabia** | **125219 (97367 , 164175)** | **1076.6 (859.2 , 1312.7)** | **156031 (122635 , 199355)** | **662.3 (541.1 , 805.5)** | **-38.5 (-51.5 , -18.4)** | **-1.68**  **(-1.75, -1.61)** |
| **Sudan** | **1390550 (797599 , 2402354)** | **3979 (2496.7 , 6601.5)** | **380930 (256106 , 552654)** | **951.9 (691.8 , 1271.9)** | **-76.1 (-85.7 , -62.8)** | **-4.82**  **(-5.07, -4.57)** |
| **Syrian Arab Republic** | **266767 (196026 , 368054)** | **1385.3 (1075.7 , 1801.9)** | **79826 (60138 , 102141)** | **659.7 (507.6 , 835.4)** | **-52.4 (-65.8 , -34.2)** | **-2.54**  **(-3.33, -1.74)** |
| **Tunisia** | **159741 (117655 , 217650)** | **1592.5 (1227.6 , 2090.4)** | **41698 (31643 , 54752)** | **386.1 (294.2 , 503.8)** | **-75.8 (-84.1 , -64.3)** | **-4.78**  **(-4.97, -4.60)** |
| **Turkey** | **2455229 (1774867 , 3365227)** | **3325.7 (2466.3 , 4501)** | **309439 (248357 , 367147)** | **415.3 (339.1 , 488.7)** | **-87.5 (-91.2 , -83.1)** | **-6.89**  **(-7.42, -6.37)** |
| **United Arab Emirates** | **7565 (6054 , 9503)** | **1316.6 (880.7 , 1565.6)** | **19275 (14480 , 26407)** | **728.2 (496.9 , 888.8)** | **-44.7 (-56.2 , -25.9)** | **-2.01**  **(-2.61, -1.40)** |
| **Yemen** | **966218 (619526 , 1646844)** | **3777.3 (2581.7 , 5947)** | **363836 (243983 , 529232)** | **1175 (834.6 , 1563.3)** | **-68.9 (-80.5 , -53.3)** | **-3.94**  **(-4.27, -3.60)** |
