# Supplementary figures and images for: The burden of lower respiratory infections and their underlying etiologies in the Middle East and North Africa region, 1990–2019: results from the Global Burden of Disease Study 2019
Source: BMC Pulm Med. 2023 Jan 4;23:2. doi: 10.1186/s12890-022-02301-7 (PMC9811697; doi:10.1186/s12890-022-02301-7)

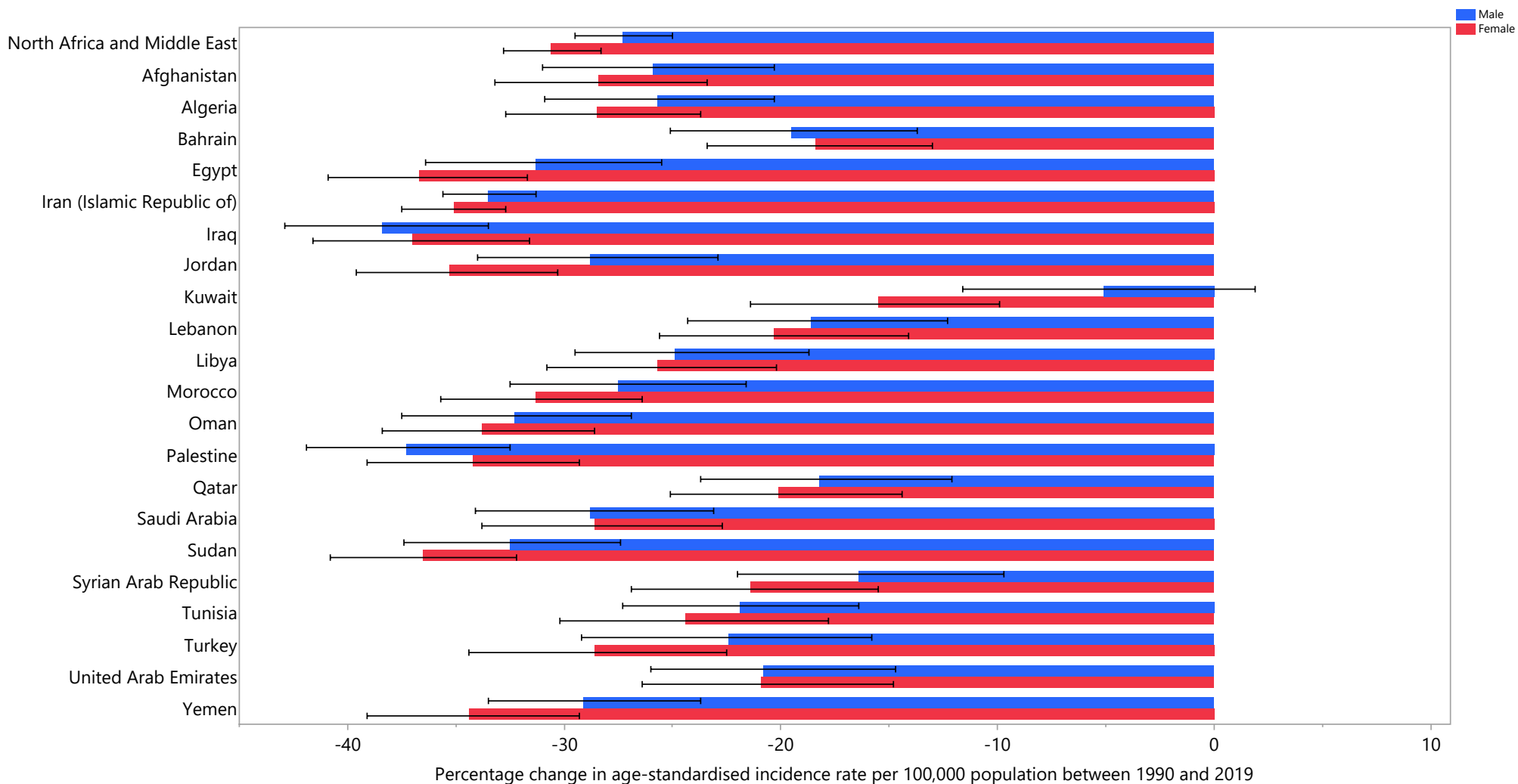

Supplement: Supplementary file 4 — Additional file 4. Figure S1: The percentage change in the age-standardised incidence of lower respiratory infections in the Middle East and North Africa region from 1990 to 2019, by sex and country. (Generated from data available from http://ghdx.healthdata.org/gbd-results-tool). [file 12890_2022_2301_MOESM4_ESM.pdf]

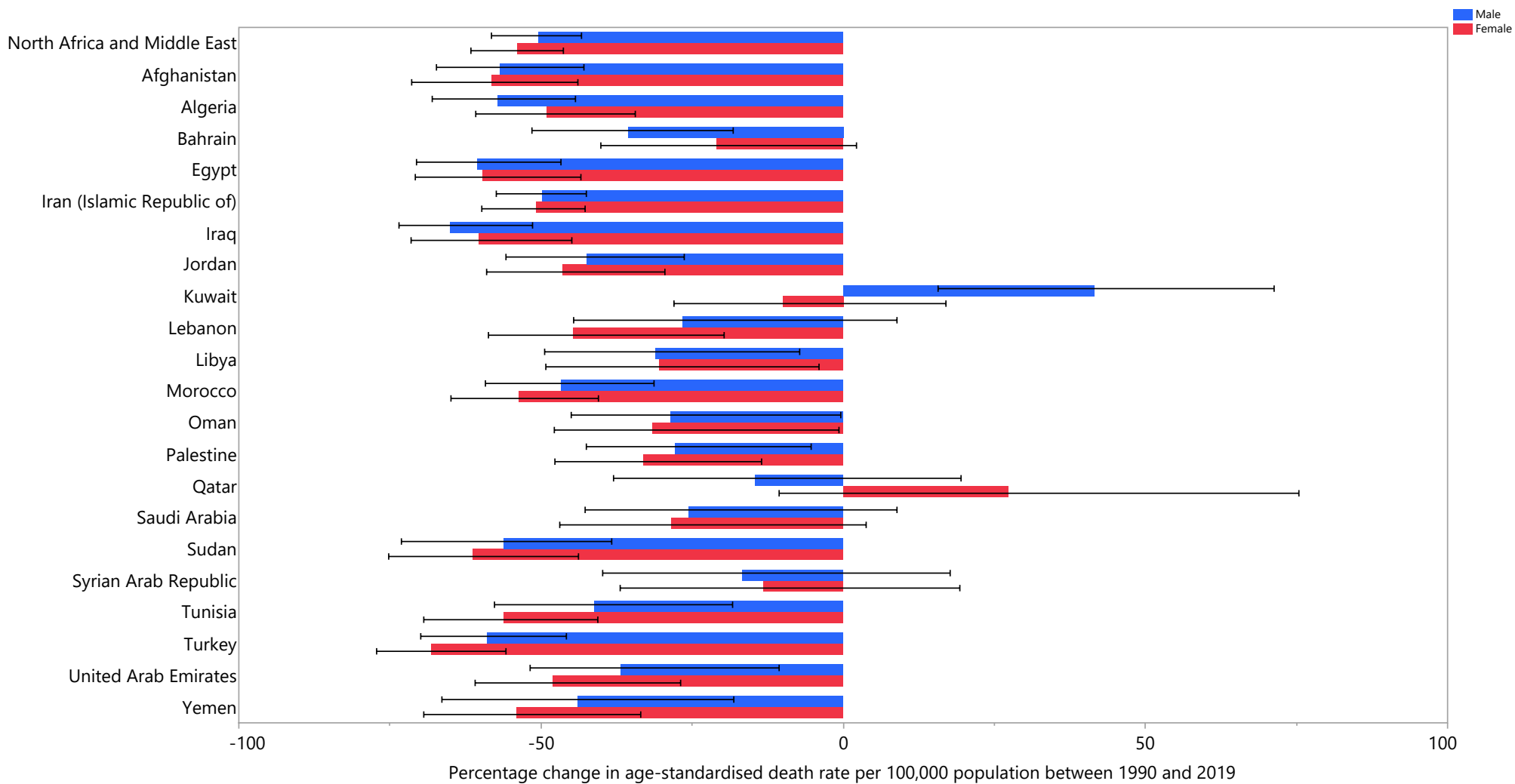

Supplement: Supplementary file 5 — Additional file 5. Figure S2: The percentage change in the age-standardised death of lower respiratory infections in the Middle East and North Africa region from 1990 to 2019, by sex and country. (Generated from data available from http://ghdx.healthdata.org/gbd-results-tool). [file 12890_2022_2301_MOESM5_ESM.pdf]

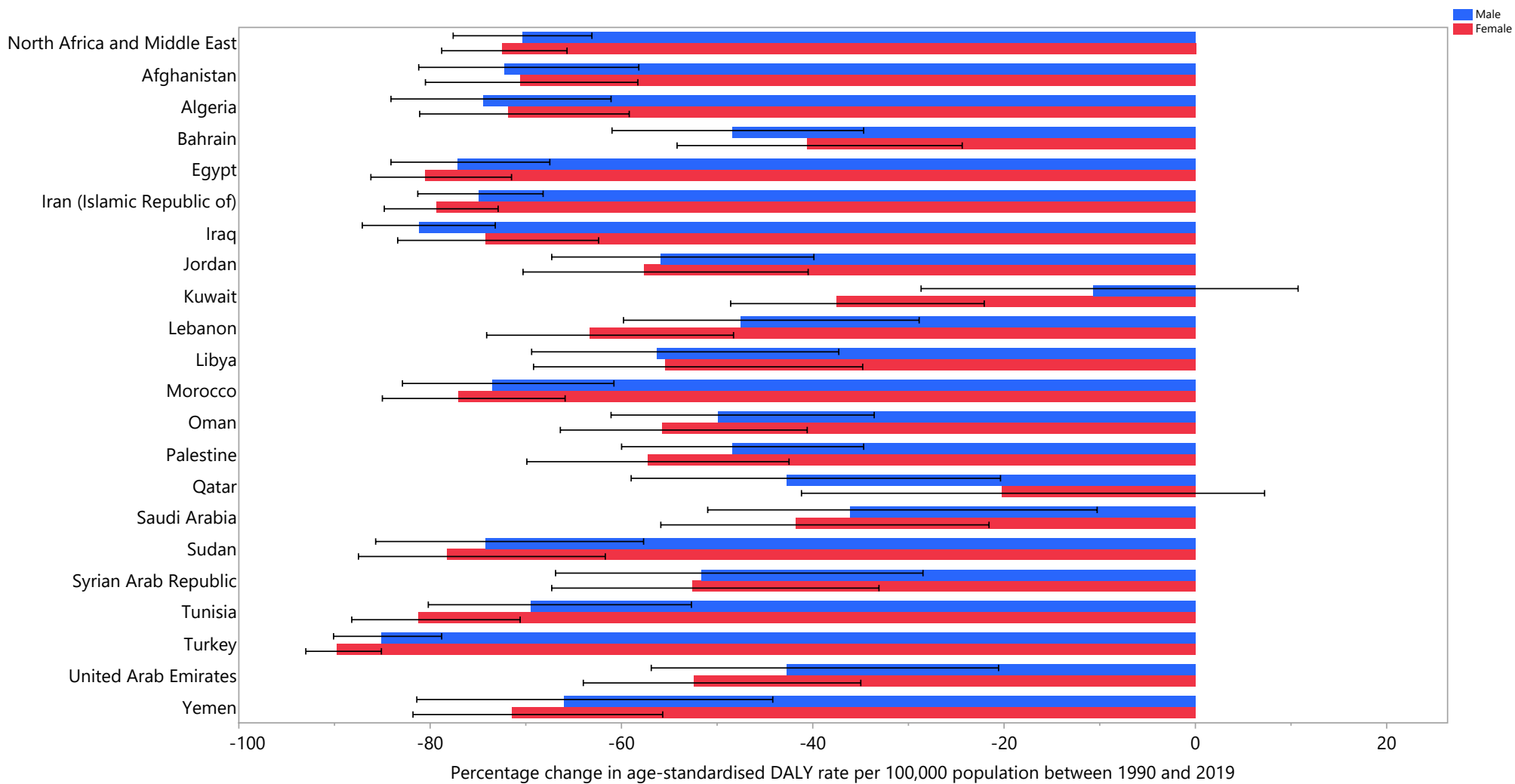

Supplement: Supplementary file 6 — Additional file 6. Figure S3: The percentage change in the age-standardised DALYs of lower respiratory infections in the Middle East and North Africa region from 1990 to 2019, by sex and country. DALY= disability-adjusted-life-years. (Generated from data available from http://ghdx.healthdata.org/gbd-results-tool). [file 12890_2022_2301_MOESM6_ESM.pdf]
